# Manometric and Sonographic Examination of Anal Sphincter Complex, Fecal Incontinence and Quality of Life Outcomes in Fournier’s Gangrene Survivors †

**DOI:** 10.3390/life16091557

**Published:** 2026-09-16

**Authors:** Özgen Işık, İrem Zehra Acar, Murat Şen, Abdullah Boğa, Ahmet Ali Aktaş, Levent Tezcan, Ersin Öztürk, Tuncay Yılmazlar

**Affiliations:** 1 Department of General Surgery, School of Medicine, Bursa Uludağ University, Bursa 16059, Turkey; 2 Department of General Surgery, School of Medicine, Acıbadem Mehmet Ali Aydinlar University, Istanbul 34752, Turkey; 3 Department of General Surgery, Medicana Bursa Hospital, Bursa 16110, Turkey

**Keywords:** Fournier’s gangrene, fecal incontinence, endoanal ultrasound, anal manometry

## Abstract

Fournier’s gangrene (FG) often requires repeated aggressive debridement. Its long-term effects on continence, anal sphincter integrity, and quality of life remain insufficiently defined. Among 188 patients treated for FG between 2000 and 2021 and assessed for eligibility, 21 survivors who had perianal debridement within 5 cm of the anal verge, healed without permanent stoma, and completed the study protocol were included. Participants underwent standardized physical examination, anorectal manometry, endoanal ultrasonography, and questionnaire-based assessment of fecal incontinence and quality of life. Majority of patients were male (66.7%) with a median age of 59 years (39–74). The median follow-up time was 83 months (27–211). On anorectal examination, 3 patients had anal fistula, 1 had anal fissure, 1 had external rectal prolapse, and 11 had decreased anal tone on digital examination. Endoanal ultrasonography demonstrated isolated external sphincter defects in 2 patients and combined external and internal sphincter defects in 2. Anorectal manometry showed decreased resting pressure in 2 patients and decreased squeeze pressure in 11. Questionnaire-based assessment demonstrated that fecal incontinence and reduced quality of life were common among long-term survivors. Long-term survivors of Fournier’s gangrene frequently report fecal incontinence and reduced quality of life, although overt sphincter defects are uncommon.

## 1. Introduction

Fournier’s gangrene (FG) is a rare but life-threatening form of necrotizing fasciitis that primarily affects the perineal, genital, and perianal regions, typically resulting from a synergistic polymicrobial infection. The etiological origins of FG are most commonly anorectal, urogenital, or cutaneous, with anorectal sources being the predominant cause and associated with a more unfavorable clinical course [1,2,3]. The disease requires prompt diagnosis and urgent, often repeated, radical surgical debridement to halt the rapid progression of infection and mitigate systemic deterioration. Reported mortality rates vary widely, ranging from 3% to 45% [4].

The cornerstone of FG management is aggressive surgical debridement, complemented by hemodynamic stabilization and empiric broad-spectrum antibiotic therapy. The success of surgical intervention hinges on the timeliness and completeness of debridement, which may need to be repeated until all necrotic tissue is eradicated [5,6]. However, extensive debridement, especially in cases with anorectal involvement, carries the risk of iatrogenic injury to adjacent anatomical structures, particularly the anal sphincter complex (ASC) and pelvic floor musculature.

In certain cases, diversion with a protective colostomy becomes necessary—either prophylactically to prevent fecal contamination or therapeutically in the presence of anal sphincter compromise [7]. Following stoma reversal, a subset of patients may experience fecal incontinence (FI) due to perineal nerve damage, muscle dysfunction, or structural tissue loss. FI constitutes a debilitating complication that substantially impairs quality of life (QoL), yet data regarding its prevalence and characteristics in FG survivors remain scarce [8,9]. 

This study aimed to evaluate fecal incontinence (FI) and quality of life (QoL) in patients who underwent surgical debridement for Fournier’s gangrene (FG) at our institution. Specifically, we sought to investigate the long-term consequences of FG-related debridement on sphincter integrity and anorectal function through a multimodal assessment protocol. 

## 2. Materials and Methods

This retrospective cross-sectional survivorship study was conducted at the Department of General Surgery, Bursa Uludağ University Faculty of Medicine. The study protocol received approval from the institutional ethics committee (Approval No: 2022-10/20), and written informed consent was obtained from all participants prior to enrollment.

**Figure 1:**
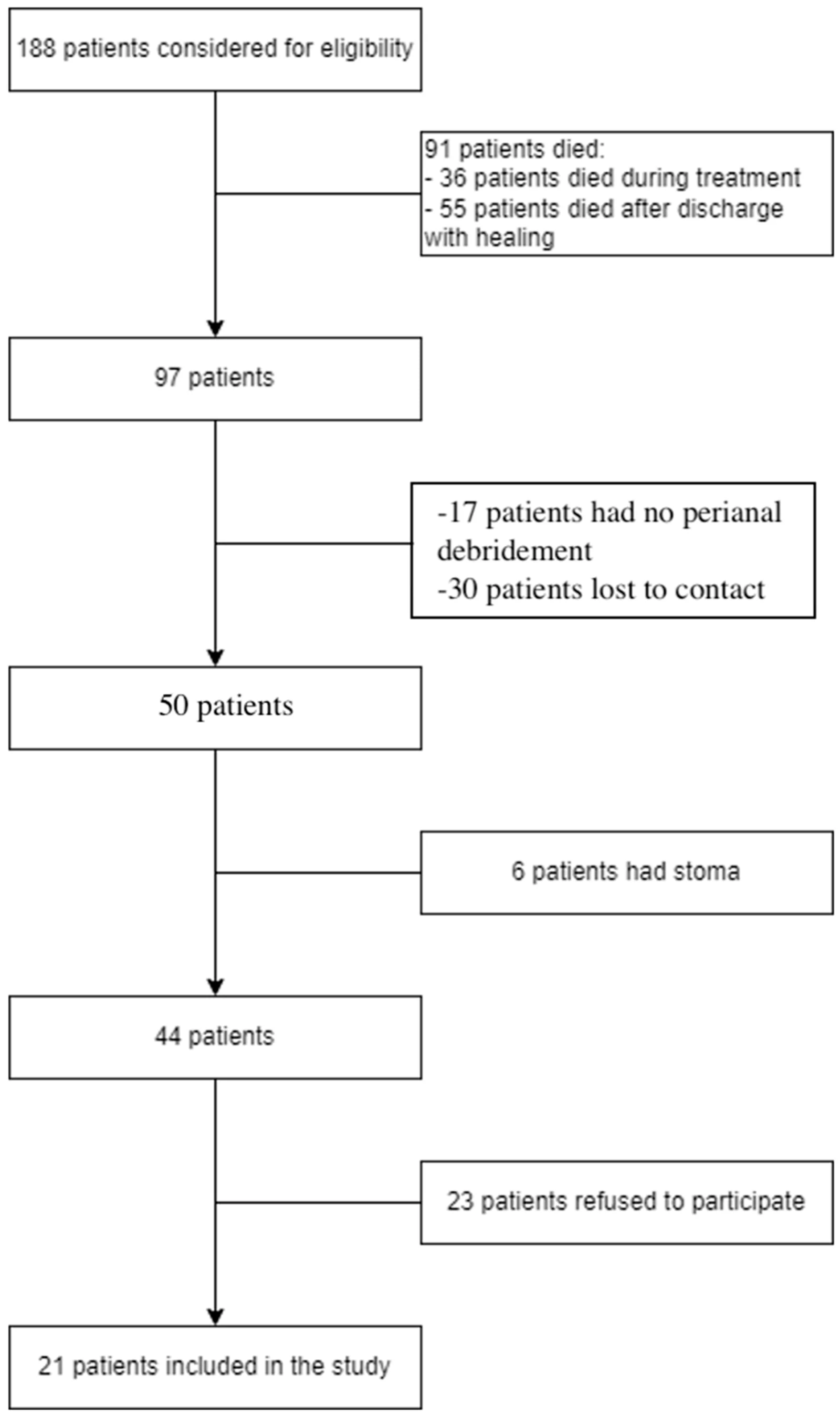
Flowchart detailing the patient recruitment process.

Patient Selection: Patients were identified from a prospectively maintained FG database comprising 270 individuals who received treatment for FG at our institution. Of these, 188 patients who underwent management between 1 January 2000 and 30 September 2021 were evaluated for eligibility. Cross-sectional functional and quality-of-life assessments were conducted between 1 January 2023 and 31 March 2023 during follow-up visits. Inclusion criteria were survival at the time of study enrollment, fulfillment of predefined clinical and procedural criteria, and the availability of valid contact information. The patient selection process, including detailed inclusion and exclusion criteria, is illustrated in Figure 1.

Inclusion criteria:

- Previous surgical debridement for FG performed at our center.
- Healing achieved without the need for a permanent stoma (restoration of intestinal continuity at least 1 year prior to functional testing).
- Debridement involving the perianal region within 5 cm of the anal verge.
- Willingness and ability to provide informed consent and participate in the full study protocol.

Exclusion criteria:

- In-hospital mortality or loss to follow-up.
- Absence of perianal debridement within the specified anatomical range.
- Current presence of a (permanent or active diverting) stoma at the time of study enrollment.
- Inability or unwillingness to provide informed consent.

Study Procedure: All eligible patients who consented to participate were thoroughly informed—both verbally and in writing—regarding the objectives of the study and the specific procedures to be undertaken. Demographic and clinical variables were systematically recorded, including sex, age at the time of surgery, current age, time from symptom onset to hospital admission, length of hospital stay, duration of intensive care unit (ICU) stay, Uludağ Fournier’s Gangrene Severity Index (UFGSI) score, presence of comorbidities, etiology of FG, anatomical extent of disease, number of debridement procedures performed, ostomy history, and methods of definitive wound closure. 

Disease severity at presentation was evaluated with the Uludağ Fournier’s Gangrene Severity Index (UFGSI). The clinical validity, predictive performance, and diagnostic utility of this scoring system specifically for patients with Fournier’s gangrene have been established and validated in the literature [10]. The UFGSI incorporates physiological and laboratory parameters (body temperature, heart rate, respiratory rate, serum sodium, potassium, creatinine, bicarbonate, hematocrit, and leukocyte count) evaluated at baseline, patient age, and disease dissemination (limited to genital region, limited to pelvis, extending beyond pelvis). A cutoff value of 9 or higher is associated with an increased risk of mortality.

Subsequent to the informed consent process, all patients were invited to complete the following validated instruments: the Short Form-36 Health Survey (SF-36) to assess overall quality of life (QoL), the Cleveland Clinic Incontinence Score (CCIS) to quantify fecal incontinence severity, and the Fecal Incontinence Quality of Life Scale (FIQL) to evaluate disease-specific QoL impact. The questionnaires were administered in their validated Turkish language versions [11,12,13]. Printed versions of all instruments were provided, and participants completed the questionnaires independently. A trained interviewer was present during completion to address any questions or clarify items as needed, thereby ensuring data reliability and response accuracy.

Assessment of Scales:

*Short Form-36 Survey:* The Short Form-36 Health Survey (SF-36) is a widely validated, generic instrument designed to evaluate both the positive and negative dimensions of perceived health status over the preceding four weeks [14]. It comprises 36 items that collectively assess eight distinct health domains: physical functioning, role limitations due to physical health problems, role limitations due to emotional problems, energy and vitality, emotional well-being (mental health), social functioning, bodily pain, and general health perception. Each domain is scored on a scale ranging from 0 to 100, where higher scores reflect better self-perceived health and functioning.

*Fecal Incontinence Quality of Life Survey (FIQL):* The FIQL Scale is a disease-specific, self-administered questionnaire designed to assess the impact of FI on patients’ daily lives and psychosocial well-being [15]. The instrument comprises 29 items distributed across four subscales: lifestyle (10 items), coping/behavior (9 items), depression/self-perception (7 items), and embarrassment (3 items). The score for each scale is calculated as the mean response to all items within that scale, obtained by summing the responses to all relevant items and dividing the total by the number of items in the scale. Higher scores indicate better functional status and quality of life. Each item is scored on a 4-point Likert scale, with response options ranging from 1 (most impaired) to 4 (least impaired), such that higher scores indicate better functional status and less impact on quality of life [15].

*Cleveland Clinic Fecal Incontinence Score (CCFIS):* Also known as the Wexner Score, it is a validated clinical tool designed to quantify the severity of FI [16]. The score ranges from 0 to 20, with a score of 0 indicating complete continence and a score of 20 representing total incontinence. For descriptive classification, a score of 0 was considered no incontinence, 1–7 mild incontinence, 8–14 moderate incontinence, 15–20 severe incontinence, and 20 complete incontinence. The scoring system incorporates five items assessing the frequency of incontinence to gas, liquid, and solid stool, the use of protective pads, and the degree of lifestyle alteration attributable to incontinence. 

Following the completion of patient-reported outcome forms, all participants underwent a standardized physical examination conducted by the same experienced colorectal surgeon to ensure consistency. The examination commenced with a visual anal inspection in the left lateral decubitus position, followed by a digital rectal examination to assess anal tone and anatomical abnormalities.

To prepare the rectum for functional and imaging assessments, patients were administered a 210 mL phosphate-based enema solution containing 28.5 g monobasic sodium phosphate and 10.5 g dibasic sodium phosphate. Post-enema, patients remained in the left lateral position for 15 min and were then instructed to evacuate the rectum twice at 30 min intervals.

High-resolution anorectal manometry (AM) was subsequently performed using a 24-channel solid-state catheter system (Medical Measurement Systems^®^, Enschede, The Netherlands; software version 9.3). The manometric protocol included the evaluation of maximum resting pressure (MxRP), maximum squeeze pressure (MxSP), the presence and adequacy of the cough reflex, and the rectoanal inhibitory reflex (RAIR). Patients were placed in the left lateral decubitus position following rectal evacuation. The catheter was inserted to position the sensor array across the anal canal and distal rectum. After a 3 min stabilization period, parameters were evaluated. Maximum resting pressure (MxRP) was measured during a 60 s resting period. Reduced resting pressure was defined as MxRP< 40 mmHg. Maximum squeeze pressure (MxSP) was measured during three consecutive maximal voluntary contractions sustained for 5 s, taking the highest value. Reduced squeeze pressure was defined as MxSP < 100 mmHg. Rectoanal inhibitory reflex (RAIR) was assessed by rapid inflation of a rectal balloon with increasing air volumes (10 to 50 mL). Cough reflex was evaluated by asking the patient to cough abruptly, assessing the reflex contraction of the external sphincter [17].

Following AM, patients underwent three-dimensional endoanal ultrasonography (3D-EAUS) using a BK Medical FlexFocus system (General Electric Healthcare^®^, Chicago, IL, USA) equipped with a 2050 transducer. The ultrasonographic assessment focused on identifying defects or discontinuities in the internal anal sphincter (IAS) and external anal sphincter (EAS), thereby enabling structural correlation with functional impairments. Sphincter integrity was evaluated along the entire length of the anal canal (deep, superficial, and subcutaneous components of the EAS, and IAS). A sphincter defect was defined as a distinct disruption or structural discontinuity in the normal hypoechoic ring of the IAS or the mixed-echogenicity muscle band of the EAS, persisting across at least two consecutive radial image slices [18].

Statistical Analysis: All statistical analyses were conducted using IBM SPSS Statistics for Windows, Version 28.0 (IBM Corp., Armonk, NY, USA). The distribution of continuous variables was assessed for normality using the Shapiro–Wilk test. Descriptive statistics were reported as medians with corresponding minimum and maximum values for non-normally distributed quantitative data and as frequencies with percentages for categorical variables.

Associations between continuous variables were evaluated using Spearman’s rank correlation coefficient, given the non-parametric nature of the data. A two-tailed *p*-value of less than 0.05 was considered statistically significant. The significance level was set at α = 0.05 for all inferential tests. To adjust for multiple statistical testing across the questionnaire correlation matrix, Bonferroni correction was applied to k = 12 primary hypothesis comparisons. Statistical significance was defined as an adjusted *p*-value < 0.05. Ninety-five percent confidence intervals (95% CIs) for Spearman correlation coefficients (r_s_) were computed using Fisher’s z-transformation.

## 3. Results

A total of 21 patients met the inclusion criteria and were enrolled in the study. The majority of the cohort was male (*n* = 14, 66.7%). The median current age at the time of evaluation was 59 years (range: 39–74), while the median age at the time of Fournier’s gangrene (FG) diagnosis and initial surgical intervention was 55 years (range: 30–69). The median duration of follow-up was 83 months (range: 27–211), allowing for a robust assessment of long-term anorectal outcomes.

Comorbid conditions were present in 15 patients (71.4%), with diabetes mellitus being the most prevalent comorbidity, documented in 10 individuals (47.6%). The most common etiology of FG was anorectal in origin (*n* = 13, 61.9%), followed by cutaneous (*n* = 5, 23.8%) and urogenital (*n* = 3, 14.3%) sources. Patients underwent a median of four surgical debridement sessions (range: 1–12), and the median duration of hospitalization was 23 days (range: 6–45). The median Uludag Fournier’s Gangrene Severity Index (UFGSI) score at admission was 7 (range: 3–10). The majority of patients exhibited Grade 2 anatomical involvement. Four patients (19.0%) had a history of diverting stoma creation, while 11 patients (52.4%) underwent tertiary wound closure. Comorbidity, hospitalization, and treatment data are presented in Table 1. Among the broader cohort of 188 patients whose medical records were reviewed for eligibility, the in-hospital mortality rate was 19.1%, consistent with the previously published literature. 

On physical examination, reduced anal sphincter tone was detected in 11 patients (52.4%) through digital rectal assessment. Notable anorectal pathologies included anal fistula (*n* = 3, 14.3%), chronic anal fissure (*n* = 1, 4.8%), and external rectal prolapse (*n* = 1, 4.8%).

The median CCIS of the study population was 5 (0–18). Assessment of fecal incontinence using the CCIS revealed that most patients reported some degree of fecal incontinence. Additionally, analysis of the SF-36 demonstrated reduced scores across multiple subdomains, indicating a generally diminished HRQoL among the study population. Disease-specific evaluation through the FIQL also reflected impaired outcomes, particularly in domains related to lifestyle and coping behavior.

A comprehensive summary of CCIS, FIQL, and SF-36 scores for the study cohort is provided in Table 2.

High-resolution AM was successfully performed in all 21 patients. Among the cohort, decreased resting pressure was observed in 2 patients (9.5%), while decreased squeeze pressure was identified in 11 patients (52.4%), suggesting compromised external sphincter function in a significant subset. The cough reflex could not be evaluated in one patient due to insufficient cooperation during the procedure. The median resting pressure was 68 mmHg, and the median squeeze pressure was 95 mmHg. RAIR testing demonstrated a positive response in 20 patients (95.2%), while 1 patient (4.8%) exhibited a negative RAIR response. Additionally, a diminished cough reflex was noted in one patient (4.8%).

A detailed summary of manometric findings is presented in Table 3.

EUS identified isolated defects in the external anal sphincter (EAS) in two patients (9.5%), and both were partial and subcutaneous EAS defects. Among those, one patient had mild incontinence and one patient had moderate incontinence. Combined injuries involving both the internal anal sphincter (IAS) and EAS were detected in an additional two patients (9.5%). Of those injuries were partial and superficial EAS defects, one patient had moderate incontinence and one patient had severe incontinence. Conversely, among the 17 patients with completely normal sphincter anatomy on EUS, 13 patients (76.5%) reported fecal incontinence, comprising 5 with mild, 3 with moderate, and 5 with severe incontinence, while only 4 were completely continent. No cases of isolated IAS injury were observed. Furthermore, among the three patients clinically diagnosed with anal fistula, low transsphincteric fistulous tracts were visualized in two cases, confirming the clinical suspicion.

Cross-tabulation of manometric abnormalities with CCIS revealed that both patients with reduced MxRP had severe fecal incontinence. Among the 11 patients with reduced MxSP, 5 had severe, 2 had moderate, and 3 had mild incontinence, while 1 patient was completely continent. Conversely, among the 10 patients with normal MxSP values, 1 presented with severe, 3 with moderate, and 3 with mild incontinence, whereas 3 patients reported no incontinence.

Correlation analyses demonstrated a statistically significant positive correlation between scores on the FIQL and the SF-36, suggesting that better disease-specific quality of life was associated with improved overall health perception. Conversely, an inverse correlation was observed between CCIS and FIQL scores, indicating that higher incontinence severity was associated with poorer quality of life. There was no significant correlation between UFGSI and fecal incontinence outcomes. Detailed correlation coefficients and significance values are presented in Table 4.

## 4. Discussion

This study revealed that FG survivors may experience persistent continence-related symptoms and reduced quality of life despite limited objective structural abnormalities on anorectal testing in this stoma-free cohort.

The median UFGSI score among the study participants was 7, which is in accordance with prior reports indicating lower scores among survivors, typically below the mortality-associated threshold of 9 [10]. However, UFGSI was not significantly associated with patient-reported fecal incontinence outcomes in the present cohort. This finding is clinically plausible, as UFGSI was developed primarily to estimate disease severity and mortality risk based on systemic parameters and anatomical extent rather than to predict long-term anorectal function. Consequently, patients surviving an acute episode of FG may remain at risk of functional impairment even in the absence of the highest initial severity scores.

An additional observation was the predominance of anorectal etiology in this cohort. This distribution is consistent with previously published data [1,2,3], and the high prevalence of anorectal cases may be attributed to referral patterns, as patients with perianal and rectal involvement are more frequently managed within general surgery departments. Notably, over half of the cases underwent successful tertiary wound closure, a factor that may have contributed to the limited extent of local tissue loss. This, in turn, could partially explain the absence of significant long-term structural and functional anorectal deficits among study participants [19].

Temporary diverting colostomy was required in four patients (19%), three of which were of anorectal origin. This is a clinically expected outcome, given the high likelihood of sphincteric and perirectal tissue involvement necessitating fecal diversion in such cases [7]. In the present series, the number of diverted patients was too small to determine whether prior stoma creation was associated with subsequent incontinence, fistula formation, or reduced quality of life. Nevertheless, the presence of continence-related symptoms after recovery highlights the importance of functional assessment in selected patients, particularly when symptoms persist after wound healing or stoma reversal.

The median CCIS in the study cohort was 5 (0–18), indicative of mild fecal incontinence severity [15]. Only four patients (19%) reported complete continence, underscoring the functional burden borne by FG survivors.

When compared to normative data from healthy Turkish populations, the median SF-36 scores in this cohort were lower across all subdomains—except for bodily pain [13]. This finding may be attributable to the long-term impact of FG and associated comorbidities.

As a result of EUS, the absence of an isolated IAS defect is an expected finding, considering the anatomical/vascular structure of the rectum and anal canal. Anal fistula associated with EAS defect was detected in two patients. Anal abscess and perianal fistulas play a role in the origin of FG. Although debridement is performed aggressively and over a wide area, it is mostly limited to superficial fasciae. It is possible that the anal fistulas detected in the patients are of FG origin and/or developed because of debridement. In all patients with anal fistula, the fistula was of anorectal origin, suggesting that anal fistula should be considered as a pathology that can be detected in the post-operative period, especially in this patient group.

High-resolution AM findings in this study revealed a median resting pressure of 68 mmHg and a median squeeze pressure of 95 mmHg, both within acceptable physiological limits and not indicative of overt sphincteric dysfunction [20]. However, reflex abnormalities were observed in two patients: one patient demonstrated an absent RAIR, while another exhibited a diminished cough reflex response that failed to exceed the maximal voluntary squeeze pressure. Notably, both patients had a documented history of DM, suggesting that these abnormalities may be more attributable to diabetes-associated autonomic neuropathy than to anatomical disruption secondary to FG or its surgical management [17].

When individual manometric findings were cross-tabulated against clinical symptoms, low MxRP was consistently associated with severe incontinence, whereas low MxSP showed a variable distribution across all symptom categories, including one asymptomatic individual. Furthermore, symptomatic incontinence was also observed in patients with preserved manometric squeeze pressures. Due to the small sample size, formal subgroup inferential testing was not performed, and these cross-tabulations are presented descriptively to illustrate the clinical heterogeneity in post-Fournier’s gangrene survivors. Larger prospective cohorts to formally evaluate structure–function relationships in this patient population are necessary.

The prognosis of patients with anorectal-origin FG is known to be less favorable than in those with other etiologies [1,21]. In such cases, the aggressive debridement required to achieve source control may result in structural damage to the continence mechanisms, predisposing patients to long-term fecal incontinence. In severe cases, the extent of tissue loss and neuromuscular damage may necessitate permanent fecal diversion, with profound implications for both physical function and mental health [7]. 

In addition to surgical factors, common comorbidities such as DM may independently impair fecal continence through mechanisms involving sensorimotor neuropathy and impaired tissue repair [17]. Furthermore, with the shifting epidemiology of FG toward older age groups, advancing age itself may contribute to continence impairment via age-related degeneration of pelvic floor support structures and reduced neuromuscular reserve [20]. As fecal incontinence is known to substantially compromise psychological well-being and social functioning, a comprehensive, multidisciplinary approach to long-term follow-up—including continence management, mental health support, and pelvic floor rehabilitation—may be warranted in this patient population [17].

The impact of FG on long-term quality of life remains underexplored in the literature, with only a limited number of studies addressing this important issue [22]. This gap may be attributed to several factors: the relative rarity and high mortality rate of the disease, prolonged hospital stays that may lead to psychological distress, and reluctance among patients to return for follow-up visits due to hospital-related anxiety or embarrassment. As a result, post-discharge functional outcomes—particularly fecal incontinence and psychosocial burden—are likely underreported. 

A notable strength of the present study is its use of a prospectively maintained institutional FG database, which facilitated the structured assessment of a rare disease. This study uniquely integrates patient-reported outcomes, physical examination findings, and objective anorectal physiology. In doing so, it provides a multidimensional understanding of the long-term sequelae of FG and its surgical management, with specific attention to both physical and psychosocial domains.

Nevertheless, several limitations should be acknowledged. The most prominent is the relatively small sample size. Additionally, patients with existing stomas were excluded from the study, precluding the evaluation of anatomical or physiological impairments that may be more pronounced in this subgroup. This exclusion likely underestimates the full spectrum of anorectal morbidity associated with FG, particularly in its most severe forms. The cross-sectional nature of the functional evaluations limits our ability to establish baseline pre-morbid anorectal function or track serial physiological changes over time. Selection bias remains an essential limitation of this long-term survivor cohort. Since a substantial proportion of the FG patients was excluded (Figure 1), the final cohort represents a highly selected group of healthier long-term survivors with successful wound healing and stoma reversal that may underestimate the true overall incidence and severity of anorectal dysfunction and quality-of-life impairment.

The use of fixed diagnostic thresholds for anorectal manometry based on the clinical literature represents a pragmatic clinical approach. However, because normal manometric parameters are known to vary significantly based on age, sex, and measurement equipment, these fixed cut-offs may not fully account for physiological variations across individual patient demographics. The lack of pre-morbid baseline anorectal manometry and continence scores, combined with the absence of a matched healthy control group, precludes definitive causal attribution of functional deficits exclusively to Fournier’s gangrene or surgical debridement. Pre-existing subclinical anal sphincter dysfunction or age-related structural changes cannot be completely ruled out. Additionally, potential confounding factors—including baseline diabetes mellitus status, advancing age, unrecorded obstetric trauma in female patients, and prior minor anorectal procedures—may independently influence both tissue healing capacity and anal sphincter tone. Another limitation is that post-operative follow-up durations varied among patients; although all patients reached a minimum 1-year threshold; when functional stabilization is expected, long-term adaptation mechanisms may evolve over time. Finally, while lower SF-36 quality-of-life scores were observed across multiple domains, direct statistical comparison against age- and gender-matched national normative reference data was not performed. Therefore, statements regarding quality-of-life outcomes should be interpreted as descriptive observations specific to this stoma-free survivor cohort rather than a definitive comparative impairment. Future studies incorporating baseline pre-treatment estimates, matched control arms, and normative population controls are necessary to confirm these exploratory observations.

## 5. Conclusions

Long-term fecal incontinence symptoms and reduced quality of life may occur in survivors of Fournier’s gangrene, particularly after perianal involvement and surgical debridement. However, patient-reported functional impairment was not consistently accompanied by major structural sphincter defects or marked manometric abnormalities in this cohort. These findings suggest that post-FG continence outcomes may reflect multifactorial mechanisms rather than anatomical injury alone. Larger prospective studies are required to define risk factors more clearly and to determine which patients may benefit from targeted anorectal functional assessment during follow-up.

## Figures and Tables

**Table 1:** Distribution of comorbidities, hospitalization, and treatment information in patients undergoing debridement for FG.

		*n* (%)
Comorbidity		15 (71.4)
Diabetes Mellitus		10 (47.6)
Origin		
	Anorectal	13 (61.9)
	Urogenital	3 (14.3)
	Cutaneous	5 (23.8)
Degree of involvement		
	Grade 1	5 (23.8)
	Grade 2	12 (57.1)
	Grade 3	4 (19)
History of stoma		4 (19)
Skin closure		
	Tertiary	11 (52.4)
	STSG *	10 (47.6)

* STSG: Split thickness skin graft.

**Table 2:** CCIS *, SF-36 ** and FIQL *** results of the FG patients.

Questionnaire	Parameter	Score Median (Min–Max)
**CCIS**		
	*No incontinence*, *n* (%)	4 (19)
	*Mild incontinence*, *n* (%)	6 (28.6)
	*Moderate incontinence*, *n* (%)	5 (23.8)
	*Severe Incontinence*, *n* (%)	6 (28.6)
**SF-36**		
	*Physical functioning*	80 (5–100)
	*Social functioning*	65 (12.5–100)
	*Role physical*	75 (0–100)
	*Role emotional*	66.6 (0–100)
	*Mental health*	68 (20–96)
	*Vitality*	50 (0–100)
	*Bodily pain*	100 (0–100)
	*General health*	55 (5–100)
**FIQL**		
	*Lifestyle*	3.8 (1–4)
	*Coping*	3.4 (1–4)
	*Depression*	3.7 (1–4)
	*Embarrassment*	1.6 (1–4)

* Cleveland Clinic Fecal Incontinence Score, ** Short Form-36, *** Fecal Incontinence Quality of Life.

**Table 3:** Anal manometry findings in patients undergoing debridement for FG.

		*n* (%)
Pressure measurements		
	MxRP * (mmHg) median (min–max)	68 (33–110)
	MxSP * (mmHg) median (min–max)	95 (51–247)
RAIR *		
	Positive	20 (95.2)
	Negative	1 (4.8)
Cough reflex		
	Positive	20 (95.2)
	Normal	19 (90.5)
	Low	1 (4.8)
	Unevaluable	1 (4.8)

* MxRP: maximum resting pressure, MxSP: maximum squeeze pressure, RAIR: rectoanal inhibitory reflex.

**Table 4:** Spearman correlation coefficient analysis of patients undergoing debridement for Fournier’s gangrene.

		Spearman r_s_	95% CI	*p*-Value	Adjusted *p*-Value
Incontinence Severity (CCIS *)					
**CCIS**	FIQL **—Coping/Behavior	−0.865	[−0.94, −0.69]	<0.001	<0.001
**CCIS**	FIQL—Lifestyle	−0.822	[−0.93, −0.60]	<0.001	<0.001
**CCIS**	FIQL—Embarrassment	−0.818	[−0.92, −0.60]	<0.001	<0.001
**CCIS**	FIQL—Depression/Self-perception	−0.648	[−0.84, −0.30]	0.001	0.018
**Disease Severity**					
UFGSI *** score	SF-36 Social Functioning	−0.433	[−0.73, 0.00]	0.050	0.599
**UFGSI score**	SF-36 Mental Health	−0.389	[−0.70, 0.05]	0.081	0.972
**UFGSI score**	SF-36 General Health	−0.231	[−0.60, 0.22]	0.314	>0.999
**UFGSI score**	CCIS	−0.083	[−0.50, 0.36]	0.721	>0.999
**UFGSI score**	FIQL—Lifestyle	0.195	[−0.26, 0.58]	0.397	>0.999
**UFGSI score**	FIQL—Coping/Behavior	0.165	[−0.29, 0.56]	0.475	>0.999
**UFGSI score**	FIQL—Embarrassment	0.113	[−0.34, 0.52]	0.626	>0.999
**UFGSI score**	FIQL—Depression/Self-perception	0.094	[−0.35, 0.51]	0.685	>0.999

95% confidence intervals were derived via Fisher’s z-transformation. Bonferroni correction was applied for primary correlation comparisons. * Cleveland Clinic Incontinence Score, ** Fecal Incontinence Ouality of Life Scale, *** Uludag Fournier’s Gangrene Severity Index.

## Data Availability

All data generated or analyzed during this study are included in this published article. The data that support the findings of this study are available from the corresponding author upon request.
